# Supplementary material for: MetaRibo-Seq measures translation in microbiomes
Source: Nat Commun. 2020 Jun 29;11:3268. doi: 10.1038/s41467-020-17081-z (PMC7324362; doi:10.1038/s41467-020-17081-z)
Supplement: Supplementary file 10 — Supplementary Data 7 [file 41467_2020_17081_MOESM10_ESM.zip › File2/Confidence_High_Taxonomy/184981_out.krona.html]

Javascript must be enabled to view this page.

members
magnitude
magnitudeUnassigned
count
unassigned
taxon
rank

184981\_out

3

superkingdom
2
3

976
2
phylum

class
200643
2

2
171549
order

family
171552
1

genus
838
1

1

SRS019127\_contig\_number\_2856
425941
species

family
815
1

1
816
genus

species
162156

SRS065176\_contig\_number\_25
1

phylum
1239
1

1
186801
class

order
186802
1

family
1
31979

genus
1

SRS1041136\_contig\_number\_15899
1485
